# Conceptualising spirituality for medical research and health service provision

**DOI:** 10.1186/1472-6963-9-116

**Published:** 2009-07-13

**Authors:** Michael B King, Harold G Koenig

**Affiliations:** 1 Department of Mental Health Sciences, University College London Medical School, Royal Free Campus, Rowland Hill Street, London, UK; 2 Duke University Medical Center, Geriatric Research, Education and Clinical Center, Veterans Administration Medical Center, Durham, North Carolina 27710, USA

## Abstract

The need to take account of spirituality in research and health services provision is assuming ever greater importance. However the field has long been hampered by a lack of conceptual clarity about the nature of spirituality itself. We do not agree with the sceptical claim that it is impossible to conceptualise spirituality within a scientific paradigm. Our aims are to 1) provide a brief over-view of critical thinking that might form the basis for a useful definition of spirituality for research and clinical work and 2) demystify the language of spirituality for clinical practice and research.

## Background

A consideration of patients' spirituality is now regarded as an important component in compassionate service delivery in a number of medical specialities in the United Kingdom[1] (see for example ) and the United States. [2-6]. However, research into the role of spirituality and health has been hampered by poorly designed studies and lack of agreement on definitions[7,8]. In this review we aim to provide a concise summary of critical thinking that might form the basis for a useful definition of spirituality for research and clinical work. We first review how the terms spirituality and religion relate to each other. We then consider varieties of spirituality and spiritual experience. Finally, we suggest that our understanding of the word spirituality must be embedded in its use by ordinary speakers rather than based on an abstraction of its meaning. In this way we arrive at a definition that might aid clinicians and researchers to address these issues.

### Religion and spirituality

Religion and spirituality were regarded as one and the same thing until recent times[9]. The advent of the twentieth century saw a gradual distinction between religion as practices and beliefs about the sacred or divine and spirituality which came to mean something more closely related to emotional experience. The psychologist William James reflected this evolution in his view of religious experience as "the feelings, acts and experiences of individual men in their solitude, so far as they apprehend themselves to stand in relation to the divine"[10]. James was influenced by Emerson's view of religion[11] when he interpreted the 'divine' as anything that is god-like[10]. In so doing, he anticipated the descriptions of spirituality that are common today. Over 50 years later Wach described spiritual (or mystical) experience as one in which there is 1) a response to a "given" that lies beyond one's everyday self; 2) total involvement; 3) a sense of something immensely real that removes for the moment everyday concerns; and 4) consequences for everyday life[12,13]. Two main points arise here. First, the definition includes characteristics as well as consequences (parts 3 and 4). Second, it disengages spiritual experience from the broader notion of spirituality and finally breaks any obligatory links with religious practice. Religion and religious practice are increasingly criticised as rigid, moralistic and unnecessary in many Western countries and the word spiritual has come to stand in opposition to them. Being spiritual has become a way of putting distance between oneself and religion, while holding onto something regarded as good. Thus spirituality is defined against what it is not. Inevitably this means that what is seen as the negative about religion will be influential in what is seen as spiritual.

### Definitions of spirituality

There are many popular descriptions of spirituality most of which are used uncritically. Even a cursory search in Google of the term *defining spirituality *reveals an array of popular definitions that share several general themes such as belief in a higher power and a sense of connectedness. There have been at least three recent attempts to define religion and spirituality for the purposes of clinical research. One was based on a traditional Roman Catholic framework[14], which limits its application to people who do not for reasons of knowledge, culture or belief understand or accept its theological basis. The second was broader in distinguishing spirituality "moored" to traditional religion from "unmoored" individualistic spirituality[15,16]:

Religion is an organized system of beliefs, practices, rituals and symbols designed a) to facilitate closeness to the sacred or transcendent (God, higher power, or ultimate truth/reality) and b) to foster an understanding of one's relationship and responsibility to others in living together in a community.

Spirituality is the personal quest for understanding answers to ultimate questions about life, about meaning and about relationship to the sacred or transcendent, which may (or may not) lead to or arise from the development of religious rituals and the formation of community

The definition of religion has held up for the purposes of research as it separates religion from its outcome in terms of health or well being. However, the conceptualization of spirituality may be too narrow in its association with what is called the 'sacred' and its stated links to religion for people who reject any such faith or understanding. The third, which arose from an extensive literature review[17] defined spirituality as "a personal search for meaning and purpose in life, which may or may not be related to religion." This definition encompassed any belief that gave meaning to life, motivated individuals, and brought "faith, hope, peace, and empowerment. "The results were joy, forgiveness of oneself and others, acceptance of hardship and mortality, a heightened sense of well-being, and "the ability to transcend beyond (*sic*) the infirmities of existence." However, the idea that spirituality is a search for meaning that has positive consequences can be criticised. First, when almost any experience can be called spiritual any attempt at definition risks becoming for all practical purposes useless. We are aware that in some religious traditions, particularly those emphasising ancestral worship, a sense of the spiritual can suffuse almost all actions or situations. However, this can also mean that if spirituality is everything then it is also nothing. Second, it focuses on self realisation and fulfilment when many regard spirituality as primarily about our relationship with others. We suggest that self realisation may be a part of spirituality but that it is too narrow to focus exclusively on this. Although we do not suggest that it must contain something that is 'other-regarding' there should at least be the possibility of it. Third, although it embraces acceptance of hardship and transcendence of the infirmities of existence, there is no consideration in it for the negative or fearsome experiences that are often described as spiritual, such as dreadful visions or an overwhelming sense of fear. Fourth, people with no spiritual belief or experience often say that they find meaning and purpose in life and thus it doesn't help us understand whether spirituality contains anything that is distinctive. For example if suicidal impulses were a regular part of everyday mood, they would have no discriminating value as a criterion for the diagnosis of major depression. Finally, it conflates spiritual experience with its outcome, in this case well-being. This distinction is crucial if there is to be any study of the consequences of spirituality.

### The universality of spiritual beliefs, practices, and experiences

People value altered states of consciousness[18] and often use music or mind altering substances to help induce liminal and ecstatic states. However, spiritual experiences and beliefs are common without such stimulation. They also occur in the absence of any religious belief or practice. A ten country ICM poll in 2004 reported that, despite falling church attendance, 67% of people in the United Kingdom professed a belief in God or a higher power. Even 30% of atheists across all countries reported that they sometimes prayed[19]. Around 90% of people in successive Harris polls in the United States profess a belief in God[20]. Surveys also show that spiritual experiences are common[12,21-24]. Whether or not such beliefs and experiences have any impact on the conduct of people's lives, however, is open to doubt[25]. There have been a large number of suggestions since the Enlightenment for why we hold spiritual beliefs or report spiritual experiences. One of the most enduring is that spiritual and religious beliefs persist because they promote social cohesion and reduce our fear of death[26,27]. Others have included the phenomenology of mental events, our human first-person experience and use of language[18,28], and natural selection of neural pathways implicated in so-called spiritual perception, presumably because it has survival value[29,30]. We shall now consider some of these suggestions in more detail.

### The phenomenology of spiritual experience

Many current approaches to understanding spiritual experience resonate with the phenomenology of Husserl and the existentialists who followed him. Consciousness and its contents contain the clues to everything that can be known about the world. Karl Jaspers' painstaking reflection on the spiritual illustrates this approach: "The fact that man senses his finiteness everywhere and cannot be satisfied with any of it points to a hidden possibility in his nature. He must have another root of his Being than that of his finiteness. If he had no pre-knowledge of the unknowable he would lack urge to enquire. But *he seeks after Being itself, after the Infinite and the Other*. Only this can give him satisfaction"[31] (Jaspers' italics). Many religious practices such as meditation, ritual, and solitude claim to move people from the verbal towards the experiential where the division between subject and object falls away [32].

That we can have any such knowledge, however, had already been challenged in the eighteenth century by Hume[33] and later Kant who argued that all our knowledge begins with experience[34]. This means that any imagined 'object' that is inaccessible to experience and that enters into no empirical relationship to an observer cannot be accessible to understanding. In the twentieth century Wittgenstein argued in his classic critique of the concept of "private knowledge" that it makes no sense to talk of "knowing" that one is in any particular mental state. Observers may know that I am in pain but I simply have my pain[35]. Wittgenstein showed that that we cannot discern the nature of a sensation purely through introspection. Rather, it is only through the grammar of our ordinary (public) psychological concepts that we can grasp the nature of a psychological state[35]. Later thinkers also denied that any knowledge can be divined from experiences in the mind that are not already part of public knowledge[18]. Thus, as we shall suggest later, the meaning of spirituality may simply reside in how we use it in language rather than in anything hidden in the minds of those who use it.

### The psychological source of the spiritual perspective

In common with much scientific thinking, the original behaviourist position[36] held that the spiritual lies outside the material world of observation. Hayes provided a riposte to this view in which he drew on behavioural analysis, phenomenology and verbal rules to suggest that our intuition of the spiritual arises from the nature of the personal perspective and our use of language[28]. As self conscious, rational creatures, we experience the world from a unique perspective. The "I-as perspective" (the observing self) has no limits and cannot be fully perceived as a "thing". In many people this "sense" of one's limitless nature may widen to the notion of the "all-perspectives" view of God. As one English theologian put it: "Perhaps the "spirit" is ... me, at the profoundest level of my being, the level at which I can no longer distinguish between what is myself and what is greater than me....where God and me mingle indistinguishably..."[37]. However, while the I-as-perspective is common to all humans, spiritual experience or belief is not. Second, while Hayes appears to agree with a long tradition reaching back to Kant[38] that the self (or soul) cannot be perceived as a thing, he nevertheless avers that it can be sensed as 'space' without any evidence of how this is possible. Third, we cannot know how the I-as-perspective might compare in other living creatures.

### Biological explanations for spirituality

We have already referred to the suggestion that a biological capacity for spiritual belief may be selected for in evolution. There have been several attempts to explain spiritual perception or discernment in biological terms. Gillespie et al[39] and Hamer[40] claimed that self-transcendence (defined as a set of personality characteristics such as feeling connected to the world and a willingness to accept things that cannot be objectively demonstrated[41]) may be heritable. We also know something of the brain function underlying the emotions joy, ecstasy, rage or fear[24,42,43], all of which may be part of ecstatic experience. Recreational drugs such as cocaine and amphetamines have actions similar to known neurotransmitters and may lead to experiences that mimic the spiritual[12]. Undoubtedly, biological structures and processes underlie all our cognitive or emotional processes. However, examining those that are involved in spiritual awareness depend on us defining it in the first place.

### The place of belief

Despite its experiential nature, spirituality often seems to require a framework or act of (usually religious) belief. This framework consists of the symbols or interpretations which Jaspers[31] considered useful only so long as they do not become concrete truths. He was critical of the paths taken by world religions as he believed concrete symbols of faith obscured spiritual understanding. However, like William James before him, he wrote during a period when spirituality and religion were increasingly regarded as distinct. One of us recently developed an instrument in England, arguably one of the most secular of developed societies, to measure spiritual belief regardless of its religious context[24]. We found that people spoke of spirituality in terms of their relationships with important others and with the world, and their beliefs about ultimate meaning. Although spirituality was often seen as a part of religious belief, there was also much discussion about spirituality separated from religion. These findings led us to develop an instrument in which 10 of out of 20 final statements described spirituality as distinct from religion[24]. Thus, it would seem that spirituality can be distinguished from religious belief and practice but whether this is the case in all parts of the world remains to be seen.

### The role of attitudes and environment

An open attitude to the *possibility *of spiritual perception may be important for it to occur[44]. Seeing involves knowing what to look for. Thus a familiar food or everyday object may take on new meaning and induce a sense of wonder when perceived closely and without judgement[45]. Intense experiences of a spiritual type may occur during prolonged periods of isolation, physical deprivation or emotional stress. Spiritual awareness is also said to arise from contemplation of works of art or intense concentration on a task, such that the separation between subject and object becomes less apparent. This includes retreat and religious worship and ritual. Ecstatic mystical experiences may occur spontaneously but periods of intense reflection or indecision have often occurred beforehand[10]. There may also be a link between such awareness and an ability to replace the usual focus on oneself with a concern for and interest in others. Religious and secular systems of morality (in contrast to narrower concepts of moralism) concur that we flourish through our ethical and loving actions towards others[46]. This suggests that although spiritual perception is not usually the result of an effort of will, certain states of mind may favour its appearance. It also suggests that spirituality is a response to the world. Rather than cultivation or improvement of an illusory self[47,48], it may involve being moved by what is other than oneself. It is communication of something within a relationship, an interaction.

### Spirits

So far we have made no mention of spirits or the spirit world, something that would have been regarded as incomprehensible in times past and even today by many people[49]. The original meaning of spirit referred to the supernatural domain of God or gods, souls, angels or demons. The human spirit was that element that enlivened the material body. Moslems and Hindus take the spirit world very seriously. A central tenet in Christianity is the gift of the Holy Spirit which is considered to bring one fully into being without displacing the self. In contrast, evil spirits may "take over" the executive function of peoples' bodies and lives, usually causing considerable unpleasantness as they do so. They are regarded as making the person less fully himself. Although obviously of importance in theological terms, there are also researchers who take some of these ideas seriously today, although most couch them in psychic rather than spiritual terms (e.g. Journal of the American Society for Psychical Research). Nevertheless, it is striking how rarely spiritual forms and beings feature in popular reviews of spirituality, given that they are so fundamental to most modern religions. Although rational enlightenment thought and the scientific progress it has brought with it have reduced much of our magical and superstitious thinking, it has also led to a hesitation to discuss such issues for fear of losing scientific respectability[49]. We return to this difficulty when we discuss the sources of spirituality.

### The self

Many forms of spirituality assume a Cartesian concept of the self as a substance that can be built up and developed in of itself[35]. Several of the definitions of spirituality we referred to earlier contain core elements of self-fulfilment and development; the realisation of a more complete person. This is particularly apparent in personal forms of spirituality in which inner observation, reflection and meditation are the main or only focus. However, the Cartesian self has been criticised ever since Kant[48]. Modern concepts of the self regard it something that emerges as we use language and relate to others[50]. A major strand of sociological thought emphasises that we come to know or construct ourselves through the actions and reactions of others[51]. Concepts of the self based on cognition and neurophysiology are also tied closely to an understanding of our perception of, and interactions with, others[52,53]. Thus, rather than objectifying and building up a self, spirituality may be better seen as relational in nature.

### A definition of spirituality

How might this brief review of such a vast and complex field help us arrive at a working concept of spirituality? We have argued that humans have always sensed a transcendent spiritual world and although throughout most of history this was placed firmly within a religious framework or narrative, since the Enlightenment there have been many alternative claims and counter claims about its nature and origins. These have included the phenomenology of our mental experiences, possible biological and evolutionary origins, the role of mind altering substances and the nature of the self. To start with, we suggest that the definition of religion already proposed by one of us[15,16] is simple and pragmatic and we shall not attempt to refine it. However, before defining spirituality we must emphasise three points. First, this very brief overview indicates that although the content of religion and spirituality may vary widely, its form is more limited. Second, although we can describe secular sources in people who understand spirituality purely in those terms, we can say nothing at all if the source is claimed to be sacred, diabolical or divine[54]. Third, it is an error to include proximate consequences such as a moral life or more distal outcomes such as better physical or mental health in the definition as this renders tautological research into consequences. Several published definitions contain within them what we would regard as its consequences [12,13,17]. Given these caveats, we propose a definition that is rooted in how we believe the word is used by everyday speakers. We suggest that the word spirituality has been difficult to define because most attempts to do so have tried to abstract the word from its application. We are guided by Wittgenstein's critique of the abstraction of the meaning of words from "the spatial and temporal phenomenon of language"[55]. Attempts to drill down to some base or 'thing' that is spirituality will founder in incomprehensibility. Rather we suggest that the best approach to the meaning of spirituality lies in how it is used in language rather than in anything hidden in the minds of those who use it[56]. With this orientation in mind, we propose in table 1 four components of spirituality, any one of which may stand alone. These components do not constitute a hierarchy of value from basic to advanced spirituality, nor is one component a bridge to the next. However, the components are ordered in terms of increasing awareness of relationship to something that is beyond empirical verification. In using the words domain and existence we do not imply a world of spirits; we emphasize again that any reference to a source of spirituality is not part of the definition (table 1). Furthermore, we suggest that desire for understanding; wonderment at beauty, art or nature; or the intention to live an ethical life are neither necessary nor sufficient for the definition as all occur in what is regarded as every day, secular experience. We do not regard the emotion in component 3 as a consequence of spirituality when it forms the route of awareness itself. When we speak of spirituality an essential part of what we mean is our emotional response. Finally, we assume that these components of spirituality are mediated by processes rooted in brain function.

**Table 1:** A definition of spirituality

**Components of the definition**	**Description**
1. *Belief*	An assent to or conviction about a domain or existence that goes beyond the material world. This includes all manner of religious or other beliefs that are not based on materialism.
	
2. *Practice*	Spiritual or religious practice at this level occurs *without *conscious awareness of, or relationship to, the spiritual realm addressed. Although it involves exercises of imagination and desire such as contemplation, prayer, reading or reflection, the self is not moved by any direct experience of relationship with or connection to the other.
	
3. *Awareness*	There is an awareness of being moved intellectually and/or emotionally. It includes contemplation, prayer, meditation or reflection when there is conscious awareness of, or response to, this dimension.
	
4. *Experience*	A discrete experience which may include diffusion of the mind, loss of ego boundaries and a change in orientation from self towards or beyond the material world. The experience usually comes unbidden but may follow a period of reflection, meditation, stress or isolation. Ecstatic experiences are of this type, but experience may be much less intense and more prolonged.
	
**Factors *not *a part of the definition**	
*Sources*	Any consideration of the source of spirituality, be it secular, sacred, divine or diabolical.
*Consequences – positive or negative*	These may be proximate such as happiness, fear, a new sense of meaning or the intention to live an ethical life; or distant such as economic success or failure and changes in physical or mental health, or in relationships.
*Other*	Secular systems of virtue, ethics or morality.

### Grounding the definition

Apart from no belief or awareness at all, there are clearly nine possible combinations of these spiritual components, two or three of which are fairly common. Component 1 alone is seen in surveys when people say they believe in God or a higher power but experience no other form of spirituality. Also very common are people who report components 1 and 2 but who have never had an awareness of a spiritual realm. People who report only component 2 are those who follow a spiritual or religious practice not because of belief or awareness but through convention or tradition. Others may report being moved in relation to a spiritual domain (components 3 and/or 4) but in the absence of specific belief. Still others encounter component 4 when they abruptly experience loss of ego boundaries and a feeling of unity without any previous spiritual belief, involvement in religion, or awareness. The distinction between awareness and experience is that the latter occurs without conscious encouragement – it is something that happens to the person. Finally, spirituality may be fluid and complex. It was reported that Mother Teresa of Calcutta's loss of awareness (component 3) did not change her beliefs or practices (components 1 and 2) but caused her considerable distress.

### Implications of this definition

We are interested in provoking debate about whether our suggestion that spirituality is used in these ways is widely recognised. Our experience as well as the evidence from qualitative research would suggest that it is[57]. Categorising individuals' accounts may help to impose structure on their complexity and to understand how spirituality is expressed. Nevertheless, we make three caveats. First, we should be wary of treating the components as if they exist materially in the minds of individuals. Second, we should avoid squeezing people's accounts into specific categories when there is no precise fit. Finally, we should regard this taxonomy as a means of understanding spirituality and not as an end in itself. A number of clinical applications arise from this definition. Most importantly, clinicians might explore the four components of spirituality and how they impact on their patients' care. We suggest a number of questions on spirituality (table 2). The first five are the most basic, while the sixth and seventh may be posed when relevant. Examples of research questions that might be pursued are: 1) What is the prevalence and stability of these four components of spirituality in patients, the general population and between cultures? 2) Are health and social outcomes different in those who regard the source as sacred or secular? 3) Is the frontier between psychotic experiences and beliefs and components 3 and 4 of our definition to be found in the phenomena described or their consequences?

**Table 2:** Questions to explore spirituality in a clinical setting

***Question***
1. Are you in any way a spiritual person?

2. Do you observe a religion?

3. Is your spirituality mainly about you personally or is it found more in your relationships with other people?

4. Does your spirituality help you cope with life's difficulties?

5. Does your spirituality help you cope with illness or disability?

6. Have you ever been aware of a spiritual realm or presence?

7. Have you ever had a very intense experience (unrelated to drugs or alcohol) in which you felt some deep new meaning in life, or at one with the world or universe? (If you believe in God it may have felt like an experience of God.)

## Conclusion

We are aware we run the risk of pleasing no-one in this very short attempt to review current thinking on spirituality, discuss the reasons why people have spiritual beliefs, and provide a definition of spirituality that can be useful to health practitioners. Limitations on space mean that we have not always been able to provide a full philosophical and conceptual argument to support all of the positions we take in this paper. As white male Christians, we are also aware that our attempt is influenced and limited by conscious and unconscious biases arising from of our own cultural and religious traditions. This said, our intention was to present an accessible, reasoned background for health researchers and for health professionals who encounter patients' (spoken and unspoken) spiritual concerns in their day-to-day work. We do not propose this definition as a finished product but with the aim of stimulating debate. We concur with others[54] that research into the role of spiritual experiences and religious belief in health, which does not stray into attempting to demonstrate that they have utility[58], is grounded in nature and does not purport to test theological or mystical mechanisms, is important and worthy of support.

## Competing interests

The authors declare that they have no competing interests.

## Authors' contributions

MK had the original idea for the paper and wrote the first draft. MK and HK authors contributed to subsequent drafts and both authors approved the final manuscript.

## Pre-publication history

The pre-publication history for this paper can be accessed here:


